# Analysis of molecular diversity, population structure and linkage disequilibrium in a worldwide survey of cultivated barley germplasm (*Hordeum vulgare *L.)

**DOI:** 10.1186/1471-2156-7-6

**Published:** 2006-01-24

**Authors:** Lyudmyla V Malysheva-Otto, Martin W Ganal, Marion S Röder

**Affiliations:** 1 Institute of Plant Genetics and Crop Plant Research (IPK), Corrensstr. 3, 06466 Gatersleben, Germany; 2 TraitGenetics GmbH, Am Schwabeplan 1b, 06466 Gatersleben, Germany

## Abstract

**Background:**

The goal of our study was a systematic survey of the molecular diversity in barley genetic resources. To this end 953 cultivated barley accessions originating from all inhabited continents except Australia were genotyped with 48 SSR markers. Molecular diversity was evaluated with routine statistics (allelic richness, gene diversity, allele frequency, heterozygosity and unique alleles), Principal Coordinate Analysis (PCoA), and analysis of genome-wide linkage disequilibrium.

**Results:**

A genotyping database for 953 cultivated barley accessions profiled with 48 SSR markers was established. The PCoA revealed structuring of the barley population with regard to (i) geographical regions and (ii) agronomic traits. Geographic origin contributed most to the observed molecular diversity. Genome-wide linkage disequilibrium (LD) was estimated as squared correlation of allele frequencies (r^2^). The values of LD for barley were comparable to other plant species (conifers, poplar, maize). The pattern of intrachromosomal LD with distances between the genomic loci ranging from 1 to 150 cM revealed that in barley LD extended up to distances as long as 50 cM with r^2 ^> 0.05, or up to 10 cM with r^2 ^> 0.2. Few loci mapping to different chromosomes showed significant LD with r^2 ^> 0.05. The number of loci in significant LD as well as the pattern of LD were clearly dependent on the population structure. The LD in the homogenous group of 207 European 2-rowed spring barleys compared to the highly structured worldwide barley population was increased in the number of loci pairs with r^2 ^> 0.05 and had higher values of r^2^, although the percentage of intrachromosomal loci pairs in significant LD based on *P *< 0.001 was 100% in the whole set of varieties, but only 45% in the subgroup of European 2-rowed spring barleys. The value of LD also varied depending on the polymorphism of the loci selected for genotyping. The 17 most polymorphic loci (PIC > 0.80) provided higher LD values as compared to 19 low polymorphic loci (PIC < 0.73) in both structured (all accessions) and non-structured (European 2-rowed spring varieties) barley populations.

**Conclusion:**

A global population of cultivated barley accessions was highly structured. Clustering highlighted the accessions with the same geographic origin, as well as accessions possessing similar agronomic characters. LD in barley extended up to 50 cM, and was strongly dependent on the population structure. The data on LD were summarized as a genome-wide LD map for barley.

## Background

Barley is one of the agronomically most important large-genome cereals with a high number of varieties and accessions worldwide. The systematic evaluation of the molecular diversity encompassed in barley genetic resources is a prerequisite for its efficient exploitation in breeding as well as for development of the strategies for optimal conservation of genetic diversity.

During the past five years an extensive amount of data was produced concerning the evaluation of genetic diversity with SSR markers for different crops, such as wheat, barley, sorghum, tomato, potato, rice and maize. An overview of the reported results for barley [1-8] and wheat [9-15] indicated that diversity parameters varied significantly between the studies. Obviously, they depended on the number of investigated loci and the number and origin of accessions involved. We selected approximately one thousand cultivated barley accessions originating from various geographic areas worldwide with different growth habits (spring/winter/intermediate), end use qualities (malting/feed/human food) and form of the spike (two-/six-row). They were genotyped with 48 SSR-markers homogenously distributed over the whole barley genome. The resulting database included approximately 45.000 datapoints, and presented genotyping information for all barley accessions coded in a binary (1/0) matrix. Genomic diversity was estimated according to the following criteria: (i) allelic richness, i.e. number of the detected alleles, (ii) gene diversity computed according to Nei [16], (iii) occurrence of unique alleles and (iv) occurrence of heterogeneous loci. The conducted genome-wide genetic diversity survey allowed to analyze the structure of this barley population. The applied set of 48 mapped microsatellite markers permitted to detect in the 953 cultivated barley accessions a total of 799 different alleles. To the best of our knowledge, this is the first time that such a comprehensive evaluation of the genetic diversity of barley germplasm from all over the world with the description of this high number of alleles and gene diversity is reported.

The development of high throughput genotyping techniques promoted publications on the possible application of association studies based on linkage disequilibrium to plant species [17-19]. In the presence of linkage disequilibrium, it is possible to identify genetic regions (if LD extends for the distance of several centiMorgans) or genes (if LD decays quickly, in few thousands of bp) associated with a particular trait of interest by genome-wide scans for genetic regions or by individual SNPs or SNP haplotypes within a candidate gene [20]. However, the data concerning the general pattern of LD in different plant species are quite scarce and very diverse. Most of the studies described LD in maize and Arabidopsis, besides single reports on LD in sugarcane, sorghum, aspen, pine and barley [17,18,21-23]. The extent of genome coverage in these studies varied from short distances as few hundred base pairs up to genetic regions as huge as tens of centiMorgans or genome-wide. To evaluate genome-wide as well as intrachromosomal patterns of linkage disequilibrium, the genotyping data produced in our study were subjected to linkage disequilibrium analysis. The estimates of LD were calculated both at the level of a diverse group of accessions originating worldwide, and within a more defined subgroup of accessions released in a specific geographic area. As a result we constructed a low-resolution (tens of centiMorgans) map of "islands" of significant LD in the barley genome which could serve as a frame for upcoming association studies in barley.

## Results

### Description of molecular diversity

The genotyping of 953 cultivated barley accessions representing geographical regions worldwide (Table 1) with 48 SSR loci allowed to identify a total of 799 alleles. The data for microsatellite loci diversity are summarized in Table 2. Alleles for each locus were present in regular one or two base pair steps. For five loci, Bmac0032 (1H), Ebmac0788 (4H), Bmag0613 (6H), GBMS0083 (7H) and Bmag0135 (7H), null alleles, that is absence of amplification products of the respective marker, were observed. The allelic richness ranged from 5 (HVM65, 6H) to 33 (Bmac0032, 1H) alleles per locus with on average 16.7 alleles per locus. The gene diversity computed according to Nei (1978) varied from 0.38 (GBMS0111, 7H) to 0.92 (Bmac0040, 6H), with the average value of 0.86. There was a high correlation coefficient between gene diversity and allelic richness (r = 0.515) as was previously demonstrated for wheat [10-14].

**Table 1:** Geographical origin of the barley accessions represented in the database.

Origin of the accessions	No. of accessions analysed
**EUROPE**	**565**
Germany	180 (141 breeder's varieties)
France	117 (83 breeder's varieties)
Great Britain	65 (42 breeder's varieties)
Russia	38
Netherlands	30 (17 breeder's varieties)
Denmark	27 (19 breeder's varieties)
Sweden	23 (12 breeder's varieties)
Czech Republic/Slovakia	13
Austria	11 (2 breeder's varieties)
Italy	8 (1 breeder's variety)
Hungary	7
Former Yugoslavia	7 (2 breeder's varieties)
Finland	5
Spain	5
Other countries	29 (1 breeder's variety)
**NEAR EAST**	**51**
Syria	10
Iran	8
Iraq	7
Israel	1 (1 breeder's variety)
Turkey	14
Jordan	5
Oman	3
Lebanon	3
**AMERICA**	**145**
Peru	13
USA	55 (3 breeder's varieties)
Canada	31
Mexico	12
Chile	6
Bolivia	6
Ecuador	9
Uruguay	6
Columbia	5
Brasil	2
**AFRICA**	**26**
Egypt	5
Libya	4
Morocco	12
Algeria	5
**ASIA**	**166**
Japan	20
India	24 (3 breeder's varieties)
China	25 (1 breeder's variety)
Nepal	18
Korea	18
Former Soviet Union Republics	38
Afghanistan	6
Pakistan	5
Bhutan	12

**Table 2:** Microsatellite loci diversity in the developed database.

**Locus**	**Chromosome**	**Allelic richness (AR)**		**Gene diversity (GD)**	**Number of unique (rare) alleles**		**Heterogeneity, (%)**
					
		**per locus**	**per chromosome**		**per locus**	**per chromosome**	
Bmac0032	1H	33	110	0.86	4 (4)	39	1.7
HVM0020		12		0.73	4 (2)		1.2
Bmag0211		13		0.8	2 (1)		1.5
Bmag0382		14		0.64	5 (3)		1.5
Bmag0718		13		0.79	3 (1)		0.5
GBMS0184		13		0.77	2 (1)		2.7
Bmag0579		12		0.59	2 (5)		0.5
GBMS0247	2H	18	106	0.83	3 (2)	33	2.2
HVM0036		19		0.8	6 (3)		1.9
Bmag0518		21		0.8	3 (4)		1.7
GBMS0229		12		0.6	2 (5)		2.0
GBMS0160		15		0.75	2 (1)		1.3
HVM0054		12		0.78	1(0)		2.0
Bmag0749		9		0.74	1(0)		0.7
EBmac0705	3H	20	167	0.58	5 (3)	70	5.2
Bmag0603		25		0.85	7 (5)		1.5
GBMS0046		30		0.82	8 (8)		2.0
HVM0060		18		0.79	3 (5)		1.4
Bmag0225		22		0.91	2 (4)		1.8
GBMS0189		27		0.81	6 (5)		2.5
Bmag0013		25		0.82	4 (5)		1.3
HVM0040	4H	14	103	0.71	3 (3)	28	1.9
EBmac0906		11		0.77	4 (1)		1.5
GBMS0087		17		0.47	5 (1)		2
EBmac0701		17		0.84	1 (1)		2.4
GBMS0133		13		0.69	2 (2)		1.5
EBmac0788		20		0.84	1 (3)		1.6
HVM0067		11		0.73	0(1)		2.3
GBMS0032	5H	14	90	0.66	1 (1)	24	2
EBmac0684		10		0.73	1 (2)		0.6
GMS0061		7		0.71	0(1)		1.4
GMS0027		32		0.8	6 (7)		3.9
GBMS0119		14		0.74	0(1)		3.3
GMS0001		13		0.59	3 (1)		2.6
Bmac0316	6H	26	127	0.74	0 (11)	28	2
GBMS0083		14		0.84	1 (1)		2.1
GBMS0125		6		0.54	1 (2)		0.7
HVM0065		5		0.59	0 (0)		0.4
EBmac0602		21		0.73	1 (3)		2.3
Bmag0613		27		0.91	2 (2)		2.1
Bmac0040		28		0.92	2 (2)		2.5
GBMS0192	7H	12	96	0.71	1 (3)	33	10.4
GBMS0035		6		0.69	0 (0)		2.4
GBMS0111		15		0.38	2 (7)		0.5
Bmag0507		18		0.85	1 (2)		2.6
EBmac0755		20		0.74	5 (4)		6.6
GBMS0183		10		0.69	2 (2)		1.2
Bmag0135		15		0.78	1 (3)		0.8
**In total**		799			121 (134)		0.8
**Average**		16.7		0.74	2.6 (2.9)		

**Figure 1:**
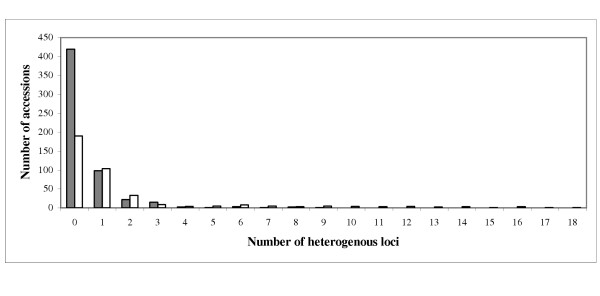
**Structure of heterogeneity detected in 953 barley accessions**. The columns represent heterogeneity in 565 European accessions (in grey) and in 388 accessions from other geographic regions (in white).

A varying percentage of heterogeneity was detected at all loci, starting from 0.4% with four heterogeneous accessions at locus HVM65 (6H) and up to 10.4% or 99 heterogeneous accessions at locus GBMS0192 (7H). However, only three loci, Ebmac0705 (3H), Ebmac0755 (7H) and GBMS0192 (7H), were heterogeneous in more than 5% of all investigated accessions. Approximately two thirds of all accessions (609 accessions) were completely homozygous. The majority of heterogeneous accessions (201 out of 344 accessions) was heterogeneous by only one locus (Fig. 1). Heterogeneity by four or more loci was observed only in 62 accessions. The most heterogeneous accession, Monte Cristo (India), carried 18 heterogeneous loci. This structure of heterogeneity was detected in both European and non-European accessions (Fig. 1).

In total, 46 out of 48 genomic loci carried alongside with the often occurring alleles also unique alleles present only once in the whole set and rare alleles, which occurred with a frequency of up to 0.5% corresponding to 4 out of 953 accessions of the set. Only two loci, HVM65 (6H) and GBMS0035 (7H), contained neither unique nor rare alleles. Altogether 121 unique and 134 rare alleles were detected comprising 32% of the available diversity.

### Geographical analysis of diversity

The 953 barley accessions included in the investigation comprised 328 modern spring and winter barley varieties obtained from various barley breeders with 320 varieties originating from Europe and two accessions of *H. vulgare *ssp. *spontaneum*. The remaining 625 accessions were chosen from the barley core collection of the genebank at Gatersleben [24] with origins from Europe (245), Asia (166), America (145), Africa (26) and the Near East (51) (Table 1). Though most of the investigated germplasm originated from Europe (565 accessions) the observed number of total alleles in European accessions (541 alleles) was comparable to the other regions (Table 3). Especially high numbers of alleles in relation to the number of investigated accessions were found for Asia, with 610 alleles in 166 accessions, and the Near East, with 503 alleles in 51 accessions. These findings are also reflected in the gene diversity also called polymorphism information content (PIC), where the European accessions averaged 0.64 (varying from 0.14 to 0.89). Accessions from other continents displayed higher values for gene diversity ranging from 0.78 for the Near East to 0.74 for Asia. The only exception was Africa with a gene diversity of 0.62, however, here the number of analyzed accessions was very low (26 accessions).

**Table 3:** Molecular diversity of the investigated accessions related to their geographical origin.

Geographic origin (number of analysed accessions)	AR*		GD*	Unique alleles, (%)	No. of accessions carrying unique alleles, (%)	Continent-specific alleles, (%)	Heterogeneous datapoints (%)	No. of accessions carrying heterogeneous loci, (%)
Europe (565)	541		0.64	20 (3.7%)	19 (3.4%)	42 (7.8%)	0.97%	146 (25.8%)
America (145)	495		0.75	12 (2.4%)	10 (6.9%)	32 (6.5%)	1.1%	48 (33.1%)
Africa (26)	311		0.62	8 (2.6%)	7 (26.9%)	16 (5.1%)	14.5%	21 (80.7%)
Asia (166)	610		0.74	39 (6.4%)	31 (19.4%)	101 (16.6%)	2.9%	97 (58.4%)
Near East (51)	503		0.78	29 (5.8%)	23 (45.1%)	51 (10.1%)	7.5%	32 (62.7%)
Total worldwide (953)	799		0.86	108 (13.5%)	90 (9.4%)	242 (30.2%)	2.1%	344 (36.1%)

*abbreviations as in Table 2.

About one quarter of the European accessions carried heterogeneous loci (Table 3), while the percentage was highest for accessions from Africa with 80.7% and the Near East with 62.7%. Related to the datapoints 0.97% of the European germplasm was heterogeneous in contrast to 14.5% of the African germplasm.

In total 277 accessions, 64 from Europe and 213 from other continents, carried unique and rare alleles, and 676 accessions of the investigated set, 501 from Europe and 175 from other continents, carried only wide-spread alleles. The highest percentage of unique alleles was observed in accessions from Asia and the Near East with 6.4 and 5.8% of unique alleles, respectively (Table 3). The total number of unique alleles in non-European accessions was 88, and they were detected at all loci. In Europe, there were 20 unique alleles at 13 loci scattered over all chromosomes except chromosome 5H. The unique alleles occurred in a total of 90 accessions with 19 accessions or 3.4% from the European pool and 71 accessions or 18.3% from the non-European pool. Two accessions of *H. vulgare *ssp. *spontaneum *included in the database carried 13 unique and 17 rare alleles. Seventeen accessions, including three European ones, carried more than one unique allele per accession (from 2 to 4).

The European and non-European germplasm shared 499 common alleles distributed over all loci. The percentage of common alleles related to the number of alleles detected for the corresponding locus varied at different loci across every chromosome in the range of 30% to 100%, with two loci, HVM65 (6H) and GBMS0035 (7H) carrying only common alleles. The remaining 300 discriminating or continent-specific alleles comprised 42 alleles occurring exclusively in European germplasm, while 243 alleles were detected only in the non-European accessions and 15 alleles were specific for *H. vulgare *ssp. *spontaneum*. Alleles specific for European accessions were registered at 22 loci, alleles specific to non-European accessions were detected at all loci except HVM65 and GBMS0035. Discriminating alleles found in specific geographic regions included unique and rare alleles, but also more frequent alleles which occurred in up to 92 accessions.

### Analysis of the population structure

**Figure 2:**
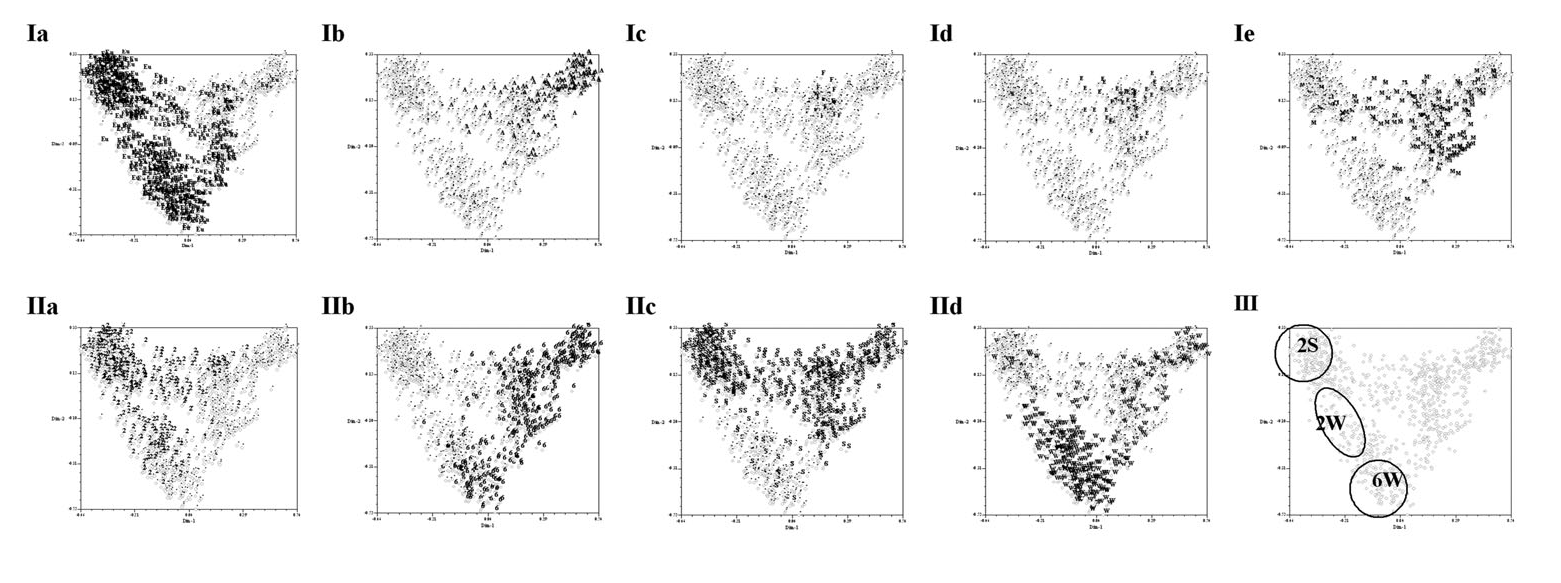
**Principal Coordinate Analysis of the global population of 953 barley accessions**. Each scatterplot presents clustering of the complete set of 953 accessions with highlighted accessions (I) originating from one geographic region, or (II) possessing a defined agronomic trait. Ia – Europe (Eu), Ib – Asia (A), Ic – Africa (F), Id – Near East (E), Ie – America (M), IIa – 2-rowed accessions (2), IIb – 6-rowed accessions (6), IIc – spring accessions (S), IId – winter accessions (W). III – Distinct clusters of European 2-rowed spring accessions (2S), 2-rowed winter accessions (2W), and 6-rowed winter accessions (6W).

Principal Coordinate Analysis (PCoA) was used to analyse substructures in the collection of barley accessions. Fig. 2 depicts two-dimensional scatterplots involving all 953 barley accessions. The first two PCoA axes accounted for 8.9% and 5.9% of the total variance. The analysis revealed distinct clustering for accessions from different continents (Fig. 2I). However, only accessions from Europe were clearly separated from the accessions originating from other continents. Clusters of accessions of non-European origin showed to a varying extent overlaps. Accessions from the Near East and Africa formed compact clusters, but were to a large extent overlayed by the cluster of Asian accessions. Accessions from America were the most dispersed, they covered almost the whole range of genetic diversity observed in non-European germplasm and had some overlap with European accessions.

Within the 565 European barley accessions no well-defined subclusters related to different countries were observed. Some tendency to form specific subgroups was observed for accessions from North European countries and the former Soviet Union (data not shown).

Clustering related to agronomic traits was performed for the traits "Growth habit" (spring/winter) and "Row number" (2-/6-row). The analysis carried out on the worldwide scale involving all investigated accessions revealed a clear separation between 2- and 6-rowed accessions, as well as a discrimination of spring and winter accessions (Fig. 2II).

The comparison of the geographically-clustered and trait-clustered scatterplots allowed to highlight the distinct groups of European 2-rowed spring accessions, European 2-rowed winter accessions, and European 6-rowed winter accessions (Fig. 2III).

### Structure of linkage disequilibrium among SSR loci

The squared allele-frequency correlations, r^2^, representing linkage disequilibrium (LD) were assessed for 964 combinations of SSR loci. In the worldwide population all intrachromosomal loci pairs were in LD with *P *< 0.001, and for 10 out of 121 evaluated loci pairs r^2 ^was higher than 0.05. In the subpopulation of 207 European 2-rowed spring barleys 45% of the intrachromosomal loci pairs were in LD witht *P *< 0.001, and for 16 pairs r^2 ^was higher than 0.05. Among 843 evaluated interchromosomal pairs of loci 98 and 42% were in LD with *P *< 0.001 in the global population and the European 2-rowed spring subpopulation, respectively, and 22 and 30 loci pairs had r^2 ^higher than 0.05. For the loci in LD with *P *< 0.001 the values of r^2 ^varied in the range of 0.0003 – 0.598 and were below 0.1 in the majority of cases. An overview of the number of loci in LD and mean LD values for loci pairs with r^2 ^> 0.05 with regard to the population structure are given in Table 4. While the percentage of loci in LD based on *P *< 0.001 decreased, the value of r^2 ^and the number of loci pairs with r^2 ^> 0.05 increased in a more uniform subpopulation such as the European 2-rowed spring barleys. This was the case for the intrachromosomal pairs of loci, as well as for loci pairs mapped to different chromosomes. The same effect of the population structure on LD, increased number of loci pairs with high values of r^2 ^along with a lower percentage of loci pairs in significant LD in subpopulations, was observed in wheat [25].

**Table 4:** Evaluation of the detected intra- and interchromosomal linkage disequilibrium (LD). LD analysis was performed for the whole set of 953 barley accessions and a subset of 207 European 2-rowed spring barley accessions. The number of common loci pairs in LD is shown in bold.

		Worldwide accessions	European 2-rowed spring accessions
Chromosome 1H	No of loci pairs in LD*	**2**+1	**2**
	Mean r^2*^	0.073	0.182
Chromosome 2H	No of loci pairs in LD*	**1**	**1**
	Mean r^2*^	0.085	0.06
Chromosome 3H	No of loci pairs in LD*	**3**	**3**+1
	Mean r^2*^	0.121	0.279
Chromosome 4H	No of loci pairs in LD*	**1**	**1**
	Mean r^2*^	0.162	
Chromosome 5H	No of loci pairs in LD*	**1**+1	**1**
	Mean r^2*^	0.1	0.19
Chromosome 6H	No of loci pairs in LD*	0	5
	Mean r^2*^		0.162
Chromosome 7H	No of loci pairs in LD*	0	2
	Mean r^2*^		0.172
Total intrachromosomal	% of loci pairs in LD with *P *< 0.001	100%	45%
	No of loci pairs in LD*	10	16
	Mean r^2^*	0.103	0.214
	Range of variation*	(0.062–0.191)	(0.053–0.598)
Total interchromosomal	% of loci pairs in LD with *P *< 0.001*	98%	42%
	No of loci pairs in LD*	22	30
	Mean r^2^*	0,064	0,083
	Range of variation*	(0.050–0.136)	(0.050–0.215)

*) Only loci pairs with r^2 ^> 0.05 at *P *< 0.001 level were evaluated.

**Figure 3:**
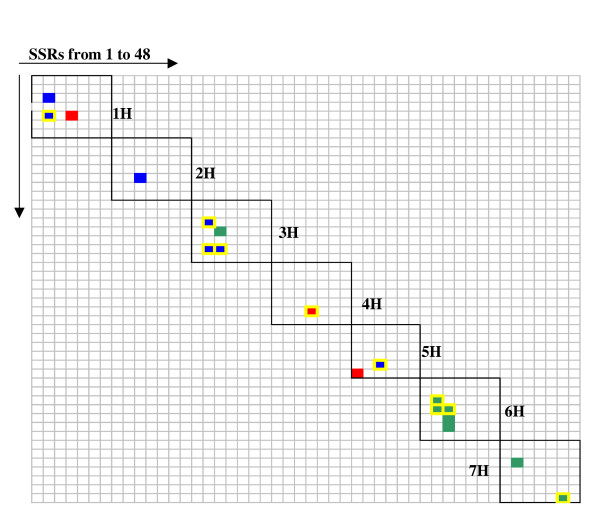
**Genome-wide map of intrachromosomal linkage disequilibrium in barley evaluated with 48 SSR loci**. Each cell represents r^2^-values for intrachromosomal loci pairs. The loci are arranged on the X- and Y-axis according to their mapping position, the order is the same as in the Table 2. White cells indicate r^2 ^< 0.05. Coloured cells indicate r^2 ^> 0.05 for the worldwide population (red), European 2-rowed spring barleys (green), and common between the two populations (blue). A yellow frame indicates r^2 ^> 0.1.

Considering all 953 accessions, the r^2 ^values for intrachromosomal pairs of loci ranged from 0.062 to 0.191 with an average of 0.103, while in the subpopulation of 207 European 2-rowed spring barleys the upper range of r^2 ^increased to 0.598 with an average of 0.214. The average values of r^2 ^for intrachromosomal loci pairs were approximately twice as high as for the interchromosomal loci pairs (Table 4). The maximum of LD between loci on different chromosomes was r^2 ^= 0.136 in analyses involving all accessions, and r^2 ^= 0.215 for the European 2-rowed spring barleys. In the worldwide barley population 10 intrachromosomal loci pairs on the chromosomes 1H, 2H, 3H, 4H and 5H, were in LD with r^2 ^> 0.05, whereas in the subpopulation of European 2-rowed spring varieties r^2 ^> 0.05 values were detected for 16 intrachromosomal pairs of loci on all chromosomes. Of those 8 loci pairs were in common between the populations. The intrachromosomal loci in LD were either moderately linked (5 loci pairs at 4–9 cM distance), loosely linked (10 loci pairs 15–50 cM apart) or independent (3 loci pairs at >50 cM distance) (classification according to [25]). The complete data concerning detected LD for all barley populations were summarized in the genome-wide map of LD for barley (Fig. 3).

**Figure 4:**
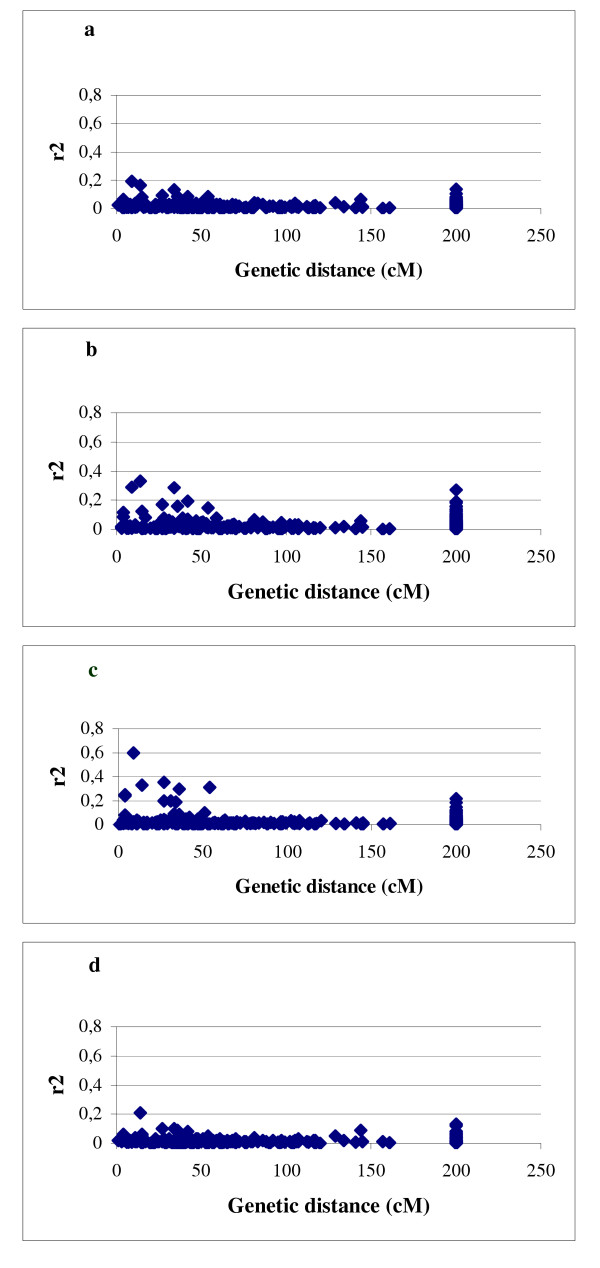
**The pattern of LD for 48 SSR loci in dependence on the population structure**. Plots of LD represented by r^2 ^against genetic distance (in centiMorgan) in the global population of 953 accesions (a), 565 European accessions (b), 207 European 2-rowed spring accessions (c), and in the random set of 200 accessions (d). Pairs of loci mapped to different chromosomes were assigned to 200 cM.

The plots of LD (r^2^) as a function of genetic distance in centiMorgans indicated a clear decay of LD with the genetic distance and also suggested its dependance on the population structure (Fig. 4a, b and 4c). Intrachromosomal LD extended to distances as long as up to 50 cM with r^2 ^> 0.05, or up to 10 cM with r^2 ^> 0.2, in the complete set of barley accessions. Elevated levels of r^2 ^were observed up to 50 cM in 565 European accessions (r^2 ^> 0.2) and in the subpopulation of 207 European 2-rowed spring barleys (r^2 ^> 0.3). To verify whether an increase of LD in the population of European 2-rowed spring varieties was a consequence of the reduced population structure and was not caused by the decrease of the number of analysed accessions, we calculated LD for a subset of 200 accessions comprising every 5^th ^accession of our set independently of the geographic origin. The resulting plot was similar to the one produced for the structured population of 953 accessions (Fig. 4d). A possible explanation for this observation may be that the European 2-rowed spring accessions were subject to uniform selection during the breeding process.

**Figure 5:**
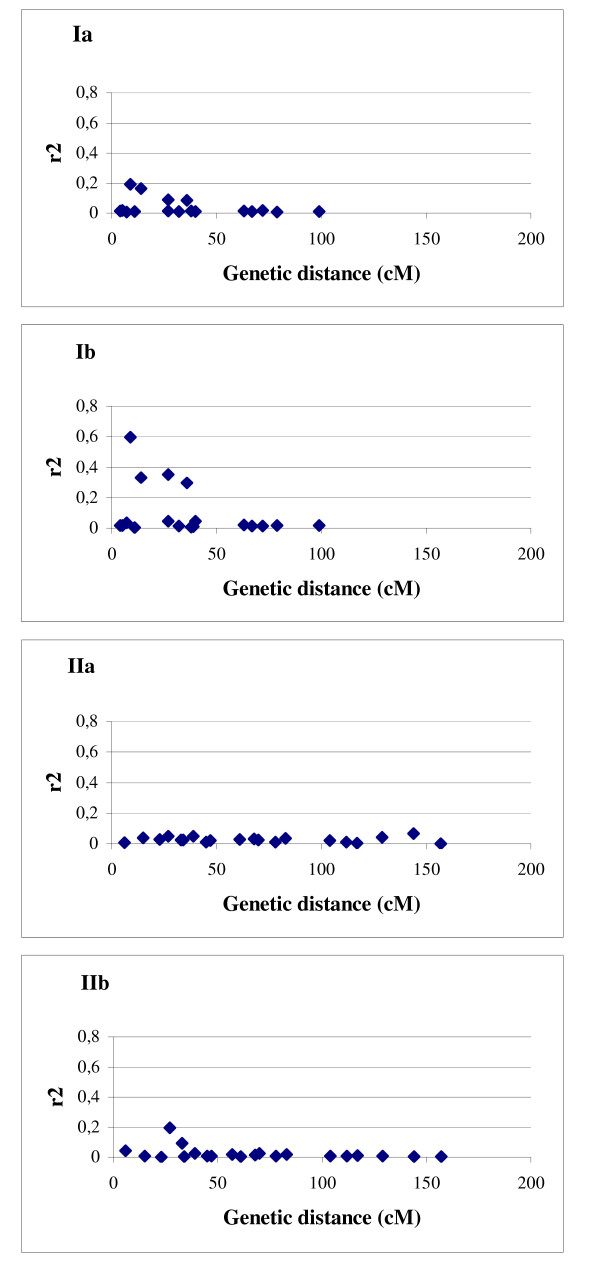
**The pattern of LD evaluated for highly polymorphic and low polymorphic SSR loci**. I – Plots of LD represented by r^2 ^against genetic distance for 17 highly polymorphic intrachromosomal pairs of loci in the global population of 953 accesions (a), and in 207 European 2-rowed spring accessions (b). II – Plots of LD against genetic distance for 19 low polymorphic pairs of loci in the global population of 953 accesions (a), and in 207 European 2-rowed spring accessions (b).

In order to test the influence of the detected polymorphism on LD we compared intrachromosomal LD estimated for the 17 most polymorphic loci (PIC > 0.80) to the 19 lowest polymorphic loci (PIC < 0.73) in both structured (all 953 accessions) and homogenous (207 European 2-rowed spring varieties) barley populations (Fig. 5I–II). While the differences between the highly polymorphic loci and the less polymorphic loci were only small in the complete structured set of 953 barley accessions (Fig. 5Ia and 5IIa), 4 additional cases of LD > 0.2 were observed only for the high polymorphic loci in the subset of 207 European 2-rowed spring accessions (Fig. 5Ib).

**Figure 6:**
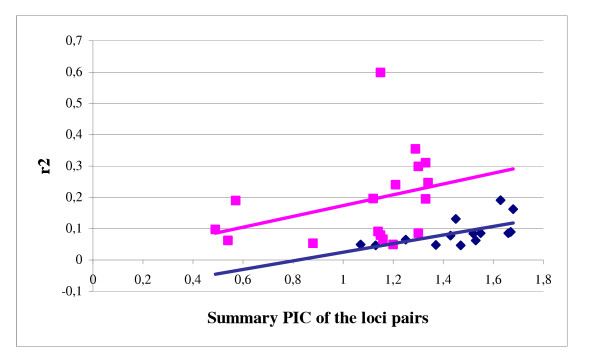
**Dependence of LD on gene diversity (PIC) and population structure**. Plots of LD represented by r^2 ^against the sum of PIC values in the global population of 953 accesions (blue), and in 207 European 2-rowed spring accessions (pink). Only intrachromosomal pairs of loci with r^2 ^> 0.05 at *P *< 0.001 level were evaluated.

The plot of r^2 ^versus the sum of PIC values for intrachromosomal loci pairs with r^2 ^> 0.05 summarized the dependence of LD on the gene diversity (= PIC) and the structure of the population (Fig. 6). Both in the worldwide population and in the European 2-rowed spring subpopulation an increase of r^2 ^values for the loci with higher gene diversity was observed. The rank correlations were moderate, however, significant and equalled r_S _= 0.705 (*P *< 0.01) and r_S _= 0.478 (*P *< 0.05) for the worldwide population and European 2-rowed spring subpopulation, respectively.

## Discussion

### Geographic origin explains the highest percentage of molecular diversity

**Figure 7:**
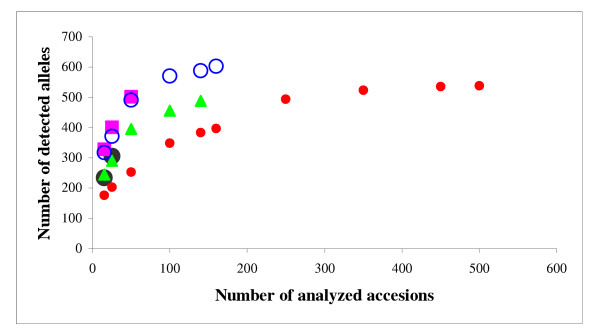
**Dynamics of the allelic richness in dependence on the number of investigated accessions**. The curves represent:  – Near East,  – Asia,  – Africa,  – America,  – Europe.

Molecular diversity in barley accessions from various geographic regions worldwide differed with respect to allelic richness, frequency of unique alleles and extent of heterogeneity (Table 3). The common alleles between Europe and other geographic regions, 499 in total, probably, represent a kind of "core" alleles for barley. However, in our study the number of accessions from different continents included in the set varied significantly. To account for the input of the number of investigated accessions into the allelic richness we drew the graph relating the number of registered alleles to the size of the investigated set of accessions within every continent (Fig. 7). The number of detected alleles increased very steeply with the increase of the set up to 100 accesssions. Beyond that the slope was more moderate. A comparison of the resulting curves for different continents clearly distinguished between three groups with a specific "diversity accumulation index". The first group representing accessions from Asia and Near East, yielded the highest number of alleles for the same number of accessions along the whole range of the set. The curves for the second group representing accessions from America and Africa displayed less allelic richness across the whole range in comparison to the first group. The third group representing European accessions was characterized by the lowest level and the most gradual increase of the number of detected alleles across the whole graph. These results indicated that the number of alleles detected for the same number of accessions at the fixed genomic loci varied in different continents. This may reflect differences in natural diversity of the species in certain geographic areas, but for justified conclusion the available data were insufficient. The results of PCoA confirmed that at the global scale, geographic origin explains the highest percentage of molecular diversity. The first principal coordinate accounting for 8.9% the of variance clearly discriminated between European accessions and accessions from other continents (Fig. 2I).

Obviously, regarding all diversity criteria applied in our study barley varieties and accessions originating from Europe possessed the lowest genetic diversity compared to other continents. This applied to the parameters allelic richness, gene diversity and percentage of unique and discriminating continent-specific alleles, as well as percentage of heterogeneity. The curves of the "diversity accumulation index" described above supported this conclusion as well. However, the pattern of clustering revealed by PCoA indicated that genetic diversity exists in the accessions from Europe which is not represented in the other continents. The European varieties formed a relatively distinct group within the diversity observed across the whole set and had little overlap with the accessions from other continents. On the other hand, accessions originating from other continents possessed genetic diversity which was not represented in Europe. It is also worth to mention that lower genetic diversity in European barleys as compared to other continents does not imply a temporal decrease of diversity due to trait-oriented breeding [4,12,14,26]. The lower genetic diversity present in European accessions may be explained by the fact that exotic varieties were very rarely involved in the breeding programmes in Europe.

In contrast to the data described for wheat [9,10,27], we did not detect any impact of the genetic factors, such as locus position on the chromosome or the motif complexity on the molecular diversity of SSR loci. However, out of seven barley chromosomes the highest diversity parameters were detected for loci on the chromosome 3H (Table 2). Varying diversity of loci mapped to different chromosomes was also registered in a set of French bread wheat accessions [14] and in three natural populations of wild emmer wheat [9].

### Genome-wide LD map for barley and its implications for association studies

We report here about genome-wide LD which extends up to 50 cM with 18 intrachromosomal loci pairs in significant LD (r^2 ^> 0.05) scattered over all seven barley chromosomes in the whole set of varieties and in a subpopulation of 207 European 2-rowed spring varieties (Fig. 3). We suggest that when evaluating LD three criteria should be considered, (i) the extent of LD, (ii) the value of r^2^, and (iii) the percentage of loci pairs in significant LD.

Genome-wide scans with RFLP loci showed a decay of LD (to values of r^2 ^< 0.05) within 10 cM in sugarcane [21] and within 50 cM in sorghum [22]. In a genome-wide scan of a global set of Arabidopsis accessions with SNPs a decay of LD for markers within genetic distances of 1 cM (250 kb) was observed, however, in several isolated local populations LD extended as far as 50–100 cM with r^2 ^> 0.2 [21,28]. Evaluation of LD in the maize genome revealed rapid decay within 1 cM up to values of r^2 ^< 0.05 when assessed with intragenic SNPs, but a much higher level of genome-wide LD when assessed with SSR loci [29]. Recently, Palaisa et al. [30] reported an extent of LD up to 600 kb in the region surrounding the maize gene *Y1 *detected with SNPs. In wild barley an excess of interlocus LD was observed by analysing 18 genes in 25 accessions, and LD levels were lower than in maize [31]. Assessment of LD with AFLP loci in the population of European 2-rowed spring barleys detected strong LD (r^2 ^> 0.7) for loci within distances of 10 cM and a decay of LD (r^2 ^< 0.1) within 100 cM [17]. Our data indicated a decay of LD within a genetic distance of 50 cM with r^2 ^> 0.05, or within 10 cM with r^2 ^> 0.2 in both a structured global population and a more homogenous subpopulation. The important conclusion out of these results is that in barley due to extensive LD the number of markers required for genome-wide association studies may be significantly lower as compared to human populations where the extent of LD usually ranges in the order of several hundreds of kb [32].

The r^2 ^values in our study were comparable to the ones reported for maize and Arabidopsis with SNPs/SSRs [28,29] and for sorghum with RFLPs [22], but were lower than detected for barley with AFLPs [17]. A possible reason is that among 121 intrachromosomal loci pairs in our set of SSRs only 17 pairs of loci were located closer than 10 cM, while most loci pairs were separated by longer distances. Recently, it was proposed that a cutoff value for useful levels of LD in plants should be fixed at r^2 ^= 0.1 [30]. We report here loci pairs in LD with r^2 ^> 0.05, highlighting on the map loci pairs with r^2 ^> 0.1 (Fig. 3).

In our study LD at *P *< 0.001 level was observed for all intrachromosomal loci pairs in the global population, and for 45% of loci pairs in the subpopulation of 207 European 2-rowed spring barleys. Interchromosomal LD with *P *< 0.001 showed 98 and 42% of loci pairs in the global population and in the European 2-rowed spring subpopulation, respectively. These are rather high percentages as compared to the reported values for 27 sorghum accessions with 8.7% of loci pairs in significant LD [22] and 10% for 102 maize accessions [29]. However, LD detected for the subgroup of 207 European 2-rowed spring accessions was comparable to about 70% of loci pairs in significant LD reported for 134 wheat varieties [25] and 60% for 146 barley varieties [17]. These data indicate that increased numbers of investigated accessions can contribute to the percentage of loci pairs in significant LD with respect to *P*-value, and that magnitude of r^2 ^is a more informative value than the percentage of loci pairs in significant LD.

Our study revealed the influence of the population structure and the polymorphism of the assessed loci on the detected levels of LD. Many research articles on LD emphasize that population stratification with unequal distribution of alleles among the groups can cause spurious associations leading to the elevated levels of LD [17,19,21]. However, as shown in our data, in cultivated barley the increase of LD (higher values of r^2^, and higher number of loci pairs with r^2 ^> 0.05) can also occur in a subpopulation with narrow molecular diversity as compared to a highly-structured population. European 2-rowed spring accessions assessed with highly polymorphic loci yielded the highest levels of LD based on r^2^, whereas the extent of LD remained similar. The reasons could be simply non-random distribution of haplotypes at the genomic level [31] which is to be expected in European 2-rowed spring accessions due to strong selection pressure. Hence, evaluation of LD should be performed in an uniform set of samples showing no population structure with selected highly polymorphic markers.

## Conclusion

A genome-wide scan with SSR loci in barley allowed to detect LD which extended up to 50 centiMorgan, and highlighted 18 genomic regions with significant values (*P *< 0.001) of r^2 ^> 0.05 scattered over all chromosomes in the whole set of varieties and in a subpopulation of 207 European 2-rowed spring varieties. The resulting genome-wide map of LD for barley had a low level of resolution accounting for the number of assessed loci per chromosome. However, these data present a frame for further association studies based on genetically mapped SSR loci.

## Methods

### Barley accessions

The seeds of 953 cultivated barley accessions were either supplied by various European breeding companies or obtained from the genebank at Gatersleben. The genebank accessions belonged to the recently established Barley Core Collection (BCC). The core collection is a set of barley genebank lines which represent the entire genetic diversity within barley germplasm [24]. Two accessions of *Hordeum vulgare *subsp. s*pontaneum *included in our set were provided by Dr. K. Pillen (University of Bonn, Germany). Several DNA samples were obtained from Dr. F. Blattner (IPK, Gatersleben). A total of 61 varieties or accessions in the database were present in duplicate or triplicate originating from different sources. The geographical distribution of investigated accessions is given in Table 1. The information concerning geographical origin, agronomic traits and pedigrees was extracted from the Catalog of barley varieties [33] or The European barley database [34].

### DNA extraction and SSR analysis

**Figure 8:**
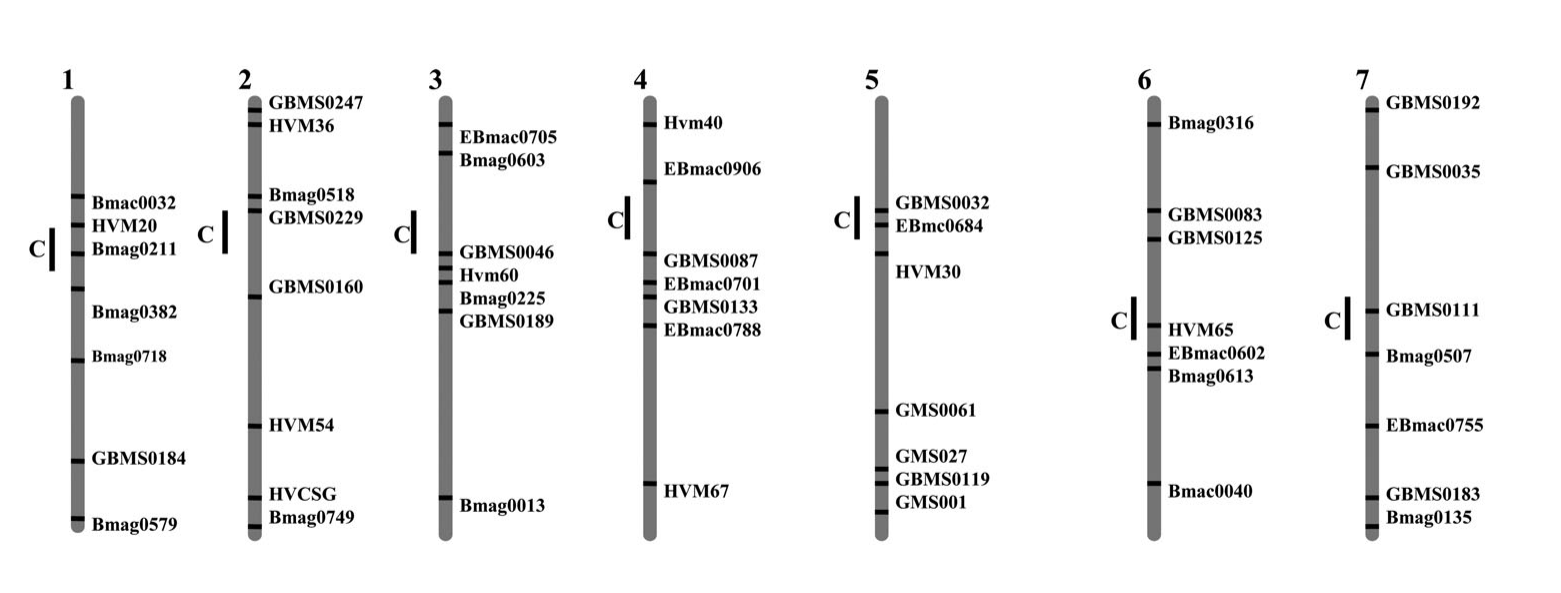
Schematic presentation of the mapped SSR loci on seven barley chromosomes.

Genomic DNA was extracted from pooled seedlings (5–10 plants per accession) as described by [35], and used as a template for the PCR assays. 48 barley microsatellite primer pairs were selected for the analysis regarding their easiness of allele calling, reproducibility and random coverage of the whole genome (Fig. 8). All markers contained dinucleotide repeats with either simple motifs (31 markers), compound motifs (10 markers) or imperfect motifs (7 markers). Primer sequences and chromosomal locations of the amplified loci were derived from Ramsay et al. [36], Liu et al. [37], Struss and Plieske [38] and from Li et al. [39]. The PCR protocol was as described by Röder et al. [40]. PCR was performed in 25 μL volume of PCR buffer (0.01 M Tris, 0.05 M KCl, 1.5 mM MgCl_2_, 0.01% gelatine) and contained approximately 100 ng of genomic DNA, 0.2 mM of dCTP, dGTP, dTTP, dATP, 0.4 μM of each primer and 1 U of *Taq *polymerase. After 3 min. at 94°C, 45 cycles were performed with 1 min. at 94°C, 1 min. at 60°C (55°C or 50°C, depending on the marker), 2 min. at 72°C and a final extension step of 10 min at 72°C. Fragment analysis was carried out using automated laser fluorescence (ALFexpress) sequencers (Amersham Biosciences, UK). Fragment sizes were calculated using the computer program Fragment Analyzer version 1.02 (Amersham Biosciences) by comparison with internal size standards, which were added to each lane in the loading buffer. Amplification products of different sizes represented different alleles. In case of two or three different alleles at the same locus, the locus was scored as heterogeneous in this accession since DNA was extracted from pooled seedlings. Scoring of multiple peaks followed the quality assurance protocol developed in the frame of EU project GEDIFLUX [41]. Namely, if the height of the additional peak/peaks was 50% or more of the height of the main peak, it was scored as "1"; if the height of the additional peak/peaks was between 50% and 10% of the height of the main peak, it was scored as "1?"; peaks with a height below 10% of the main peak were not scored. The information concerning occurrence of additional questionable alleles was included in the database, but was not used for the analysis of genomic diversity.

### Data analysis

Molecular diversity within the whole set of accessions and within two main subgroups, that is varieties released in Europe and accessions originating from other continents, was estimated according to the following parameters: (i) allelic richness as a total number of the detected alleles and a number of alleles per locus, (ii) gene diversity also called polymorphism information content (PIC) computed according to Nei [16], (iii) occurrence of unique and rare alleles and (iv) occurrence of heterogeneous loci. The level of heterogeneity for each locus was estimated as percentage of accessions carrying double or triple alleles at the corresponding locus. Alleles were considered to be unique if they occurred in one accession, and rare if they occurred in less than 0.5% of the investigated accessions, that is in up to 4 accessions. Alleles occurring in more than 0.5% of investigated accessions were referred to as wide-spread or often occurring alleles. These statistics were calculated with the programme GeneFlow V.6 (developed by GENEFLOW Inc. [42]).

The computer programme NTSYSpc 2.1 was applied to perform principal coordinate analysis (PCoA) of 953 worldwide accessions and of subsets of accessions originating from different continents using genetic similarity matrix [43] based on genetic similarity according to Nei and Li [44].

### Evaluation of linkage disequilibrium

LD between pairs of polymorphic loci mapped on the same chromosome as well as on different chromosomes was evaluated using the software package TASSEL developed by the Edward Buckler group [45]. LD was estimated by squared allele-frequency correlations (r^2^). Since SSRs are multi-allelic markers TASSEL calculates a weighted average of r^2 ^between any two loci [46] by essentially calculating r^2 ^for all possible combinations of alleles, and then the alleles' frequencies are used to weight them [45]. Since all heterogeneities were considered as missing data, the number of assessed combinations of SSR loci (964) was lower than expected (1128). The significance of pairwise LD (*P *values) among all possible pairs of 48 loci was also evaluated by TASSEL with the rapid permutations test. The loci were considered to be in significant LD if *P *< 0.001. The plots of LD (r^2^) for pairs of loci versus genetic distance in cM between loci in pair were drawn from r^2 ^values calculated by TASSEL. For the pairs of loci mapped to different chromosomes genetic distances of 200 cM were assigned.

## Authors' contributions

LMO participated in DNA extraction, carried out the SSR genotyping and prepared the database files, performed the data analysis and the statistical analysis, and drafted the manuscript. MWG conceived the study, participated in its design, collected the used barley accessions and drafted the manuscript. MSR conceived the study, participated in the database development and data analysis, drafted the manuscript and coordinated the project. All authors read and approved the final manuscript.
